# Supplementary material for: The antidepressant sertraline provides a novel host directed therapy module for augmenting TB therapy
Source: eLife. 2023 Jan 11;12:e64834. doi: 10.7554/eLife.64834 (PMC10159622; doi:10.7554/eLife.64834)
Supplement: Transparent reporting form [file elife-64834-transrepform1.pdf]

eLIFE
Westbrook Centre, Milton Road
Cambridge CB4 1YG
UK
P 01223 855340
W elifesciences.org
T @elife

# *eLife's* transparent reporting form

We encourage authors to provide detailed information *within their submission* to facilitate the interpretation and replication of experiments. Authors can upload supporting documentation to indicate the use of appropriate reporting guidelines for health-related research (see EQUATOR Network), life science research (see the BioSharing Information Resource), or the ARRIVE guidelines for reporting work involving animal research. Where applicable, authors should refer to any relevant reporting standards documents in this form.

If you have any questions, please consult our Journal Policies and/or contact us: editorial@elifesciences.org.

**Sample-size estimation**

- You should state whether an appropriate sample size was computed when the study was being designed
- You should state the statistical method of sample size computation and any required assumptions
- If no explicit power analysis was used, you should describe how you decided what sample (replicate) size (number) to use

Please outline where this information can be found within the submission (e.g., sections or figure legends), or explain why this information doesn't apply to your submission:

All infection and analyses were performed in groups and numbers according to standard established protocols. Most of the macrophage infection and downstream analyses experiments were performed in triplicate wells- technical replicates (n=3) with N=2 or 3 biological replicates for the experiments. Each replicate involved fresh/ independent stocks of cell lines and Mtb cultures. The experimental design was based on standard assay published practices. Statistical analysis involved standard T test for significance. The work does not involve any high through put sequence data. This information is provided in the legends of all figures.

**Replicates**

- You should report how often each experiment was performed
- You should include a definition of biological versus technical replication
- The data obtained should be provided and sufficient information should be provided to indicate the number of independent biological and/or technical replicates
- If you encountered any outliers, you should describe how these were handled
- Criteria for exclusion/inclusion of data should be clearly stated
- High-throughput sequence data should be uploaded before submission, with a private link for reviewers provided (these are available from both GEO and ArrayExpress)

Please outline where this information can be found within the submission (e.g., sections or figure legends), or explain why this information doesn't apply to your submission:

**Statistical reporting**

All infection and analyses were performed in groups and numbers according to standard established protocols. Most of the macrophage infection and downstream analyses experiments were performed in triplicate wells- technical replicates (n=3) with N=2 or 3 biological replicates for the experiments. Each replicate involved fresh/ independent stocks of cell lines and Mtb cultures. The experimental design was based on standard assay published practices. Statistical analysis involved standard T test for significance. The work does not involve any high through put sequence data. This information is provided in the legends of all figures.

- Statistical analysis methods should be described and justified
- Raw data should be presented in figures whenever informative to do so (typically when N per group is less than 10)
- For each experiment, you should identify the statistical tests used, exact values of N, definitions of center, methods of multiple test correction, and dispersion and precision measures (e.g., mean, median, SD, SEM, confidence intervals; and, for the major substantive results, a measure of effect size (e.g., Pearson's r, Cohen's d)
- Report exact p-values wherever possible alongside the summary statistics and 95% confidence intervals. These should be reported for all key questions and not only when the p-value is less than 0.05.

Please outline where this information can be found within the submission (e.g., sections or figure legends), or explain why this information doesn't apply to your submission:

Standard T test was used for evaluation of statistical significance. Data is represented as average of all replicates with SEM. P values less than 0.05 was considered significant. The values are represented in the individual figures. This information is provided in the Methods section as well as the figure legends of the manuscript.

(For large datasets, or papers with a very large number of statistical tests, you may upload a single table file with tests, Ns, etc., with reference to sections in the manuscript.)

**Group allocation**

- Indicate how samples were allocated into experimental groups (in the case of clinical studies, please specify allocation to treatment method); if randomization was used, please also state if restricted randomization was applied
- Indicate if masking was used during group allocation, data collection and/or data analysis

Please outline where this information can be found within the submission (e.g., sections or figure legends), or explain why this information doesn't apply to your submission:

Animals were selected in order to maintain similar numbers of both genders in any group. Animals within a group were randomly selected so as to avoid any specific bias in grouping.

**Additional data files ("source data")**

- We encourage you to upload relevant additional data files, such as numerical data that are represented as a graph in a figure, or as a summary table
- Where provided, these should be in the most useful format, and they can be uploaded as "Source data" files linked to a main figure or table

- Include model definition files including the full list of parameters used
- Include code used for data analysis (e.g., R, MatLab)
- Avoid stating that data files are "available upon request"

Please indicate the figures or tables for which source data files have been provided:

This manuscript does not include any sequencing or high through put data. Also this work does not include any protein crystallization and diffraction analysis. The manuscript does not involve data for submission to depositing datasets into a domain-specific public archive.
